# Clinical characteristics of severe neonatal enterovirus infection: a systematic review

**DOI:** 10.1186/s12887-021-02599-y

**Published:** 2021-03-15

**Authors:** Meng Zhang, Haoran Wang, Jun Tang, Yang He, Tao Xiong, Wenxing Li, Yi Qu, Dezhi Mu

**Affiliations:** 1 Department of Pediatrics, West China Second Hospital, Sichuan University, No. 20, Section 3, Renmin south road, Chengdu, 610041 China; 2 Key Laboratory of Obstetrics & Gynecologic and Pediatric Diseases and Birth Defects of the Ministry of Education, Sichuan University, Chengdu, 610041 China; 3 Department of Clinical Medicine, Sichuan University, Chengdu, 610041 China

**Keywords:** Enterovirus infection, Neonates, Severe, Lethality, Complications, Clinical characteristics

## Abstract

**Background:**

Enterovirus (EV) is a common cause of infection in neonates. Neonates are at high risk of enterovirus infection with serious clinical manifestations and high lethality. This review systematically summarized the clinical characteristics of neonates with severe enteroviral infection to provide evidence for the identification and treatment of severe neonatal EV infection.

**Methods:**

PubMed, Embase, and Web of Science were searched for original studies on neonates with severe EV infections from January 1, 2000, to November 27, 2020. Two reviewers independently screened the literature, extracted the data, and performed a descriptive analysis.

**Results:**

In total, 66 articles with 237 cases of severe neonatal enterovirus infection were included. All neonates developed severe complications. Among them, 46.0% neonates had hepatitis or coagulopathy, 37.1% had myocarditis, 11.0% had meningoencephalitis, and 5.9% had other complications such as hemophagocytic lymphohistiocytosis and pulmonary hemorrhage. The lethality rate of neonates with severe infection was 30.4%. The highest lethality rate was 38.6%, which was observed in neonates with myocarditis. In 70.5% neonates, the age at the onset of symptoms was less than 7 days. Coxsackievirus B infection was seen in 52.3% neonates. The most common symptoms included temperature abnormalities (127, 53.6%), rash (88, 37.1%), poor feeding (58, 24.5%), and respiratory symptoms (52, 21.9%). The main treatment included transfusion of empirical antibiotics (127, 53.6%), blood components (100, 42.2%), intravenous immunoglobulin (IVIG; 97, 40.9%), mechanical ventilation (51, 21.5%), and extracorporeal membrane oxygenation (ECMO; 43, 18.1%). Additionally, antiviral medications pleconaril (14, 5.9%) and pocapavir (3, 1.3%) were administered.

**Conclusions:**

Lethality was high in neonates with severe enterovirus infection, especially in those complicated with myocarditis. The most common symptoms included temperature abnormalities, rash, and poor feeding. The chief supportive treatment consisted of transfusion of blood components, mechanical ventilation, and ECMO. Empirical antibiotics and IVIG were widely used. Antiviral medications included pocapavir and pleconaril; however, more clinical evidence regarding their efficacy is needed.

**Supplementary Information:**

The online version contains supplementary material available at 10.1186/s12887-021-02599-y.

## Background

Enteroviruses affect millions of people worldwide from all age groups. According to the data from the Center for Disease Control and Prevention in America, non-polio enteroviruses cause 10–15 million infections and tens of thousands of hospitalizations in the United States each year [1]. Disease activity is typically seasonal, and most infections occur in the summer and early fall in temperate parts of the world [2]. Enterovirus infections have variable manifestations. Asymptomatic infections account for approximately 50% of the cases [3]. Symptomatic enterovirus infections range from nonspecific febrile illnesses to life-threatening diseases such as myocarditis or sepsis. The main manifestations include hand-foot-mouth disease, acute hemorrhagic conjunctivitis, herpangina, etc. [3] Infants and people with weak immune systems have a greater chance of developing severe complications.

Enterovirus is a common cause of infections in neonates. Infections in newborns may be acquired vertically before, during, or after delivery, horizontally from family members, or by nosocomial transmission in nurseries [4]. Enteroviruses can seriously affect the nervous and cardiovascular systems in neonates, resulting in myocarditis, meningoencephalitis, and other severe complications. Because of the functionally immature immune system, newborns are at high risk for the development of serious clinical manifestations of infectious diseases [5]. This study systematically reviewed the clinical characteristics of neonates with severe enteroviral infection, with the aim of gathering information for the identification and treatment of neonatal severe enterovirus infections.

## Methods

### Search strategy and selection criteria

We systematically searched for relevant studies in the following English databases: PubMed, Embase, and the Web of Science. We limited our search to English language articles published between January 1, 2000, and November 27, 2020. We also reviewed the references of all the included articles and major reviews. We combined all search records of databases using Endnote 9.1, which was used to manage and de-duplicate records. The following medical subject headings were used: “Enterovirus Infections,” “Coxsackievirus Infections,” “Echovirus Infections,” “Infant, Newborn.” The detailed search strategy for PubMed is shown in Frame s1.

The titles and abstracts of the searched citations were screened for a full-text review by two independent reviewers. Two independent reviewers evaluated full-text articles using inclusion and exclusion criteria. Any discrepancies between the reviewers were resolved by discussion with the entire review team. Studies were included if they met the inclusion criteria: (1) primary research articles, (2) study on the clinical characteristics of severe neonatal enterovirus infections. A severe neonatal enterovirus infection was defined as the presence of severe diseases such as myocarditis, meningoencephalitis, hepatitis, coagulopathy, sepsis, and other life-threatening diseases. The diagnostic test criteria for enterovirus infections included positive enterovirus cultures or positive polymerase chain reaction (PCR) testing of the patient samples, (3) study published in English language, (4) study with complete diagnosis and treatment process of severe neonatal enterovirus infections. The exclusion criteria were: (1) study designed to evaluate the risk factors or virus serotypes that included only the number or proportion of patients without clinical characteristics, (2) study about non-severe neonatal enterovirus infection, (3) study with only abstract, (4) reviews.

### Data extraction and analysis

Data extraction was performed by two independent reviewers and included epidemiological features, clinical features, and treatment. Epidemiology features extracted included the country, number of neonates according to the diagnostic criteria, outcome and prognosis, age at onset, sex, gestational weeks, birth weight, delivery mode, enterovirus infection serotype, and transmission mode. We classified and analyzed the severe cases according to the major complications. The major complications were clearly reported in the original study, which were defined by the following criteria: (1) the complication mentioned in the title of the study, (2) the major diagnosis in the study, (3) the complications that were described most detailed, (4) other complications except for the major one was found at autopsy. Two reviewers (MZ and HW) assessed the risk of bias of the included studies using the Joanna Briggs Institute (JBI) criteria [6]. The descriptive analysis was carried out using the constituent ratios (cases/total cases, %) because of the quantity limitations.

## Results

**Fig. 1:**
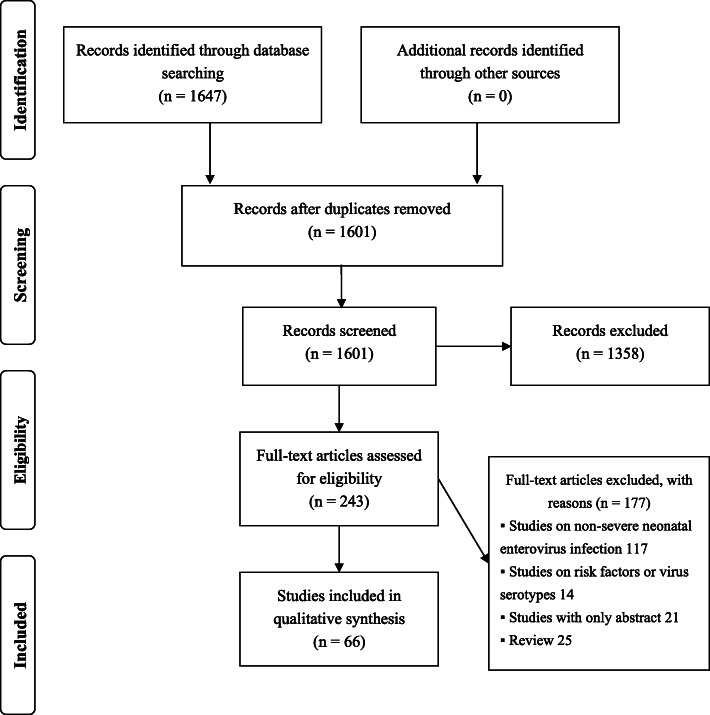
Diagram flow of studies selection

A total of 1647 studies were identified through the database search, and 243 of these were retained after screening the titles and abstracts. Subsequently, 177 studies were excluded during the full-text review. Finally, 66 articles with 237 cases of severe neonatal enterovirus infection were included in the analysis. Figure 1 shows the flow diagram of the study selection process. Table s1, s2 and s3 in the supplementary material show the general characteristics of the included neonates and the assessment of the risk of bias. The clinical characteristics of neonates with severe enteroviral infection according to the major complications were showed in Table 1.

**Table 1:** Clinical features of neonates with severe enteroviral infections (%)

	Hepatitis or coagulopathy	Myocarditis	Meningoencephalitis	Other	Total
Reference (*n* = 66)	17 (25.5)	31 (47.0)	10 (15.2)	9 (13.6)	66
Cases (*n* = 237)	109 (46.0)	88 (37.1)	26 (11.0)	14 (5.9)	237
Country (*n* = 37)	8 (21.6)	14 (37.8)	7 (18.9)	8 (21.6)	37
Sex	Male/Female: 57/46	Male/Female: 42/29	Male/Female: 11/10	Male/Female: 6/3	Male/Female: 116/88
GA (weeks)	Preterm/Full-term: 53/54	Preterm/Full-term: 26/25	Preterm/Full-term: 14/11	Preterm/Full-term: 8/6	Preterm/Full-term: 101/96
BW (g)	1735–4200	1730–4500	1100–4400	2260–4010	1100–4500
Mode of delivery	Caesarean/Vaginal: 51/50	Caesarean/Vaginal: 26/17	Caesarean/Vaginal: 11/13	Caesarean/Vaginal: 7/6	Caesarean/Vaginal: 95/86
Maternal manifestation	33 (30.3)	20 (22.7)	5 (19.2)	12 (85.7)	70 (29.5)
Days at onset <=7	98 (89.9)	35 (39.8)	20 (76.9)	14 (100.0)	167 (70.5)
Virus isolation					
Rectal/stool	77 (70.6)	16 (30.2) ^b^	18 (69.2)	7 (50.0)	118 (58.4)
Respiratory^a^	74 (67.9)	18 (34.0) ^b^	5 (19.2)	4 (28.6)	101 (50.0)
CSF	49 (45.0)	22 (41.5) ^b^	19 (73.1)	5 (35.7)	95 (47.0)
Blood	24 (22.0)	33 (62.3) ^b^	3 (11.5)	5 (35.7)	65 (32.2)
Urine	33 (30.3)	NA	1 (3.8)	2 (14.3)	36 (17.8)
Virus serotypes^b^	CVB: 71 (81.6); Echovirus: 16 (18.4)	CVB: 39 (97.5); Echovirus: 1 (2.5)	CVB: 7 (58.3); Echovirus: 4 (33.3); EV71: 1 (8.3)	CVB: 7 (63.6); Echovirus: 4 (36.4)	CVB: 124 (82.7); Echovirus: 25 (16.7); EV71: 1 (0.7)
Clinical signs					
Temperature abnormalities	82 (75.2)	24 (27.3)	16 (61.5)	5 (35.7)	127 (53.6)
Rash	73 (67.0)	NA	10 (38.5)	5 (35.7)	88 (37.1)
Poor feeding	23 (21.1)	23 (26.1)	11 (42.3)	1 (7.1)	58 (24.5)
Respiratory symptoms	11 (10.1)	24 (27.3)	5 (19.2)	5 (19.2)	45 (19.0)
Lethargy	20 (18.3)	13 (14.8)	5 (19.2)	2 (14.3)	40 (16.9)
Jaundice	32 (29.4)	5 (5.7)	1 (3.8)	NA	38 (16.0)
Circulatory failure or shock	1 (0.9)	35 (39.8)	1 (3.8)	NA	37 (15.6)
Arrhythmias	1 (0.9)	27 (30.7)	NA	1 (7.1)	29 (12.2)
Thrombocytopenia	9 (8.3)	11 (12.5)	NA	8 (57.1)	28 (11.8)
Ascites	27 (24.8)	NA	NA	NA	27 (11.4)
Hepatomegaly	19 (17.4)	3 (3.4)	NA	3 (21.4)	25 (10.5)
Poor perfusion	10 (9.2)	11 (12.5)	1 (3.8)	1 (7.1)	23 (9.7)
Irritability	7 (6.4)	1 (1.1)	9 (34.6)	NA	17 (7.2)
Seizure	1 (0.9)	4 (4.5)	10 (38.5)	NA	15 (6.3)
Hypotonia	5 (4.6)	1 (1.1)	3 (11.5)	NA	9 (3.8)
Diarrhea	NA	2 (2.3)	6 (23.1)	1 (7.1)	9 (3.8)
Other manifestation	Myocarditis: 24; DIC: 8; Encephalitis: 5; Intracranial hemorrhage: 5; Pneumonitis: 3; Renal insufficiency: 3	Meningoencephalitis: 10; Hepatitis: 7; DIC: 6; Renal failure: 3	Hepatitis: 4; Acute renal tubular necrosis, DIC, myocardial necrosis, pneumonitis: 1; Central diabetes insipidus: 1;	Table 2	NA
Auxiliary examination	AST or ALT elevated: 85 (78.0)	BNP elevated: 20 (22.7); Troponin I or T elevated: 30 (34.1); ECG: myocardial ischemia: 30 (34.1); Echocardiograph: ventricle dilation or function depression: 56 (63.6)	MRI: white matter injury: 21 (80.8); Ultrasonography: extensive periventricular echogenicity: 16 (61.5)	NA	NA
Treatment					
Systematic support	Blood component transfusions: 94 (86.2); Mechanical ventilation: 17 (15.6); Blood exchange transfusion: 4 (3.7); Peritoneal dialysis: 4 (3.7)	ECMO: 43 (48.9); Mechanical ventilation: 27 (30.7); Cardiopulmonary resuscitation: 5 (5.7)	Mechanical ventilation: 4 (15.4)	Blood component transfusions: 6 (42.9); Mechanical ventilation: 3 (21.4)	Blood component transfusions: 100 (42.2); Mechanical ventilation: 51 (21.5); ECMO: 43 (18.1)
Antibiotics	95 (87.2)	16 (18.2)	8 (30.8)	8 (57.1)	127 (53.6)
IVIG	56 (51.4)	31 (35.2)	2 (7.7)	8 (57.1)	97 (40.9)
Antivirals	Pleconaril: 9 (8.3); Pocapavir: 1 (0.9)	Pleconaril: 5 (5.7); Pocapavir: 2 (2.3)	NA	NA	Pleconaril: 14 (5.9); Pocapavir: 3 (1.3)
Organ specific treatment	Liver transplantation: 3 (2.8)	Cardiac transplantation: 4 (4.5); Mitral valve replacement: 1 (1.1)	NA	Liver transplantation: 1 (7.1)	9 (3.8)
Outcome	Survived/Died: 80/29	Survived/Died: 54/34	Survived/Died: 23/3	Survived/Died: 8/6	Survived/Died: 165/72

^a^ included throat, nasopharynx, or tracheal

^b^ Data of some neonates were not available and were not included in the calculation

*CVB* Coxsackievirus B, *EV71* Enterovirus 71, *NA* Not available, *DIC* Disseminated intravascular coagulation, *AST* Aspartate aminotransferase, *ALT* Alanine transaminase, *BNP* Brain natriuretic peptide, *MRI* Magnetic resonance imaging, *ECMO* Extracorporeal membrane oxygenation, *IVIG* Intravenous immunoglobulin

Overall, 237 neonates developed severe complications of enterovirus infection, with a male/female ratio of 116/88, and birth weight ranging from 1730 to 4500 g. In total, 51.3% (101/197) of the preterm infants and 52.5% (95/181) of the neonates were delivered by cesarean section. In 70.5% (167 cases) of the cases, the age at the onset of symptoms was less than 7 days. Seventy cases (29.5%) were associated with maternal disease before delivery, while 16 cases (6.8%) reported siblings or friends with symptoms. Maternal amniotic fluid culture revealed scarce *Staphylococcus epidermidis* in a case of myocarditis [7]. Also, a mother was diagnosed with chorioamnionitis [8] and amnionitis was suspected in another mother [9]. Enterovirus was identified mostly from the rectal or stool samples (118, 49.8%), followed by respiratory samples (101, 42.6%). Additionally, 82.7% (124 cases) of the neonates had coxsackievirus B (CVB) infection, 16.7% (25 cases) had echovirus infection, and 0.7% (1 case) had enterovirus 71 (EV71) infection. The clinical manifestations included temperature abnormalities (127, 53.6%), rash (88, 37.1%), poor feeding (58, 24.5%), respiratory symptoms (45, 19.0%), lethargy (40, 16.9%), jaundice (38, 16.0%), circulatory failure or shock (37, 15.6%), arrhythmias (29, 12.2%), thrombocytopenia (28, 11.8%), poor perfusion (23, 9.7%), irritability (17, 7.2%), hypotonia (9, 3.8%), and diarrhea (9, 3.8%). A total of 127 (53.6%) neonates were treated with empirical antibiotics and 97 (40.9%) neonates with intravenous immunoglobulin (IVIG). Antiviral medications included pocapavir (3, 1.3%) and pleconaril (14, 5.9%). Altogether, the lethality rate was 30.4% (72 cases).

A total of 109 cases of hepatitis or coagulopathy from 17 studies [9–24] were included (57 boys, 46 girls). Maternal illness in the period from 2 months prepartum to 1 week postpartum was reported in 33 cases (30.3%), including fever in 23, abdominal pain in six, flu-like syndrome in five, and diarrhea in four cases. In three cases, siblings had symptoms of a febrile illness and sore throat a few days before delivery. Neonates presented with the first symptoms between the day of birth and 1 month; 98 (89.9%) presented within 7 days of birth. Eighty-seven records of enterovirus serotypes were extracted, comprising 16 cases of echovirus infection (echovirus 3 in one, echovirus 21 in one, echovirus 11 in four, echovirus 30 in two, echovirus 7 in two, echovirus 6 in two, echovirus 5 in one) and 71 cases of CVB (CVB1 in 22, CVB2 in one, CVB3 in 46, CVB4 in one, CVB5 in one). The most common symptom was temperature abnormalities, with 60 cases of temperature instability, 17 cases of fever, and five cases of hypothermia. Other presenting symptoms were rash (73, 67.0%), jaundice (32, 29.4%), and ascites (27, 24.8%). Complications associated with hepatitis accounted for 23 cases of myocarditis, one case of hypertrophic cardiomyopathy, five cases of intracranial hemorrhage, five cases of encephalitis, eight cases of disseminated intravascular coagulation (DIC), three cases of renal insufficiency, and three cases of pneumonitis. Elevation of aspartate aminotransferase (AST) or alanine transaminase (ALT) was reported in 85 cases. A total of 95 (87.2%) neonates were treated with antibiotics and 56 (51.4%) were treated with IVIG. Antiviral medications utilized were pocapavir (1, 0.9%) and pleconaril (9, 8.3%). Moreover, 94 (86.2%) neonates required blood component transfusions, such as fresh-frozen plasma, platelets, and red blood cell concentrate. Other systematic supportive treatment included mechanical ventilation (17, 15.6%), peritoneal dialysis (4, 3.7%), hemodialysis (1, 0.9%), blood exchange transfusion (4, 3.7%), and continuous venovenous hemofiltration (1, 0.9%). Three (2.8%) neonates received liver transplants. Twenty-nine of the 109 (26.6%) neonates died and were aged between 8 days and 2 months.

A total of 88 neonates in 31 studies [7, 9, 25–53] developed the major complication of myocarditis, with a male/female ratio of 42/29. In total, 54.7% (35/64) neonates were less than 7 days old at the onset of symptoms. Twenty neonates had a history of maternal disease before delivery, while eight cases reported siblings or friends with symptoms. Thirty-nine neonates had CVB infection (CVB1 in 13, CVB2 in 2, CVB3 in 13, CVB4 in 5, CVB5 in 3, and CVB2 in 1). One neonate had an echovirus 6 infection. Clinical signs of temperature abnormalities (eight neonates with hypothermia, 15 with fever, and one with temperature instability) were observed in 24 neonates. Signs of myocarditis included respiratory symptoms (24, 27.3%), arrhythmias (27, 30.7%), circulatory failure or shock (35, 39.8%), and poor perfusion (11, 12.5%). Myocarditis was reportedly accompanied by other complications including meningoencephalitis (10 cases), renal failure (3 cases), DIC (6 cases), and hepatitis (7 cases). Elevations of troponin and brain natriuretic peptide (BNP) were observed in 30 and 20 cases, respectively. Other primary auxiliary examinations undertaken were electrocardiogram (ECG) for signs of myocardial ischemia (30, 34.1%) and echocardiography for signs of ventricle dilation or function depression (56, 63.6%). Mechanical ventilation was used in 27 neonates, and cardiopulmonary resuscitation was required in five neonates. In addition, 43 neonates received extracorporeal membrane oxygenation (ECMO) support. The medications included antibiotics (16, 18.2%), IVIG (31, 35.2%), pocapavir (2, 2.3%), and pleconaril (5, 5.7%). Moreover, four neonates survived after heart transplantation and one underwent mitral valve replacement. The lethality rate was 38.6% (34/88) in neonates with myocarditis, and 40.7% (22/54) of the survivors had sequelae or required cardiac medication.

Twenty-six cases in 10 studies [8, 54–62] were diagnosed with meningoencephalitis, with a male/female ratio of 11/10. Manifestations of meningoencephalitis in the mother and other family members were found in five and three cases, respectively. The enterovirus serotypes included four cases of echovirus (echovirus 6 in one, echovirus 30 in two, echovirus 31 in one), seven cases of CVB (CVB1 in four, CVB2 in two, CVB3 in one), and one case of EV71. Signs of central nervous system disease included seizures (10, 38.5%), lethargy (5, 19.2%), irritability (9, 34.6%), tonic–clonic movements of the upper extremities (1, 3.8%), right-sided hemiparesis (1, 3.8%), weak gag reflex (1, 3.8%), and full fontanelle (1, 3.8%). Complications of central diabetes insipidus occurred in one neonate. White matter injury was detected by magnetic resonance imaging (MRI) in 21 cases, while 16 cases exhibited extensive periventricular echogenicity on brain ultrasonography. Four neonates were treated with mechanical ventilation and two were administered IVIG. Altogether, 23 of the 26 neonates survived, with neurological sequelae reported in six.

Other rare complications are shown in Table 2, including hemophagocytic lymphohistiocytosis (HLH) [63–66], pulmonary hemorrhage [67], pulmonary hypoplasia [68], persistent pulmonary hypertension [69], bone marrow failure [70], and congenital skin lesions [71]. Among the six neonates with HLH, four developed fever, abdominal pain, and flu-like symptoms before delivery. All neonates developed the disease within 5 days. Five neonates were treated with IVIG. In the end, one neonate died and another received a liver transplant at the age of 2 months. In four cases consisting of two sets of twins with pulmonary hemorrhage, both the mothers had developed fever and other symptoms on the day of the delivery. The onset ages of the two sets of twins were 7 days and 5 days, respectively. Only one neonate survived with mild disease. There was a single case of pulmonary hypoplasia characterized by a total failure of the development of terminal respiratory units. Echovirus 11 was positive in the amniotic fluid. The neonate was delivered at 38 weeks gestational age and died one hour later. Another case involved intrauterine echovirus 11 infection with persistent pulmonary hypertension and pneumonia. The neonate died 36 h after birth. There was also a case of bone marrow failure and concomitant enteroviral infection. Bone marrow aspiration and biopsy revealed hypocellularity. The neonate finally improved. A boy with congenital disseminated papulovesicular, nodular, bullous, and necrotic ulcerated rash was also reported. He subsequently developed pneumonia, carditis, and hepatitis. CVB3 was identified in the neonate’ s serum. The boy survived with sequelae at 6 months of age.

**Table 2:** Clinical features of neonates severe enteroviral infections of rare complications (%)

	Hemophagocytic lymphohistiocytosis							
Reference	Miyoshi, 2020	Fukazawa, 2013	Watanabe, 2019	Lindamood, 2011				
Country	Japan	Japan	Italy	Canada				
Sex	Female	Male	Male	Male			Male	Female
GA (weeks)	38	35	37	37			38	41
BW (g)	2860	2260	NA	4010			3550	3120
Mode of delivery	Vaginal	Caesarean	NA	Caesarean			Vaginal	Caesarean
Maternal manifestation, days onset before delivery	Fever, 0	Abdominal pain, 1; Fever, 0	Fever, 0	Flu-like illness, 0			NA	Flulike illness, 2 weeks
Days at onset	3	4	4	3			5	0
Virus isolation (serotype)	Blood, NPS, stool, urine (CVB3)	NPS, stool (CVB1)	CSF, pharyngeal fluid, stool, urine (Echovirus 7)	Blood			Blood	Blood, CSF
Clinical signs	Apnea, fever, petechiae, poor feeding, thrombocytopenia	Apnea, hepatosplenomegaly, thrombocytopenia	Apnea, lethargy	Bleeding, fever, hepatosplenomegaly, thrombocytopenia			Cyanosis, hemodynamic instability, respiratory distress, thrombocytopenia	Lethargy, petechiae, splenomegaly, thrombocytopenia
Other manifestation	Coagulopathy, liver dysfunction	Coagulopathy	DIC	Coagulopathy, liver failure			Coagulopathy	Coagulopathy, meningoencephalitis
Auxiliary examination findings	Hyperferritinemia	Leukocytosis	Elevated LDH, AST, ferritin levels	Anemia			Anemia, elevated ferritin level	Elevated ferritin level
Treatment	Blood component transfusions, IVIG	Blood transfusions, recombinant thrombomodulin, IVIG	Blood component transfusions, mechanical ventilation, thrombomodulin, antibiotics, IVIG	Blood component transfusions, cryoprecipitate, IVIG, liver transplantation at 2 months of age			Blood component transfusions, continuous venovenous hemofiltration, cryoprecipitate, mechanical ventilation	Blood component transfusions, cryoprecipitate, IVIG
Outcome, last follow-up	Survived, 25 days	Survived, 11 months	Survived, 18 months	Survived, 2.5 years			Died, 14 days	Survived, 2 weeks
	Pulmonary hemorrhage				Bone marrow failure	Congenital Skin Lesions	Pulmonary hypoplasia	Persistent pulmonary hypertension
Reference	Orbach, 2016				Tarcan, 2001	Sauerbrei, 2000	Tassin, 2014	Willems, 2006
Country	Israel				Turkey	America	France	Belgium
sex	NA (2 twins)	NA	NA	NA	Male	Male	NA	Female
GA (weeks)	30	30	36	36	40	39	38	36
BW (g)	NA	NA	NA	NA	3800	NA	NA	2810
Mode of delivery	Caesarean	Caesarean	Vaginal	Vaginal	Caesarean	Vaginal	Vaginal	Caesarean
Maternal manifestation, days onset before delivery	Abdominal pain, seizures and fever, 0	Abdominal pain, seizures and fever, 0	Fever, a few hours	Fever, a few hours	Diarrhea and fever, 2 weeks	Mild signs of respiratory infections, 2 weeks	Spontaneous demise of a co-twin, 14 gestational weeks	Abdominal pain and fever
Days at onset	7	7	5	5	5	0	0	0
Virus isolation (serotype)	CSF (CVB3)	CSF (CVB3)	Brain, liver, lung, stool (CVB2)	CSF, stool (CVB2)	Stool (Echovirus 11)	Serum (CVB3)	Amniotic fluid, lung (Echovirus 11)	Intestine, lung, stool, tracheal aspirate (Echovirus 11)
Clinical signs	Cutis marmorata, poor response, thrombocytopenia	Cutis marmorata, hemodynamic instability, tachycardia, respiratory distress, thrombocytopenia	Cutis marmorata, fever, poor perfusion, thrombocytopenia	Fever	Diarrhea, fever, hepatosplenomegaly, petechiae, rash	Atopic dermatitis with Candida infection, bronchopulmonal dysplasia, impaired central coordination, papulovesicular, nodular, bullous and ulcerative rash	32 WG a bilateral abnormal lung development	Respiratory distress, purpura
Other manifestation	DIC, intraventricular hemorrhage	DIC	NA	NA	Transient pancytopenia concurrent	Hepatitis, myocarditis, pneumonia	NA	DIC, meningitis, pneumonia
Auxiliary examination findings	Acidosis, elevated transaminase, leukopenia	Leukopenic	NA	NA	Bone marrow aspiration and biopsy: hypocellularity	NA	Ultrasound and MRI: bilateral abnormal lung development	NA
Treatment	Antibiotics, IVIG	Mechanical ventilation, antibiotics, IVIG	Antibiotics	Antibiotics	Antibiotics, IVIG	Mechanical ventilation, antibiotics	Adrenaline, mechanical ventilation	Inhaled nitric oxide, prostacyclin infusion, antibiotics, surfactant
Outcome, last follow-up	Died, 8 days	Died, 12 days	Died, 8 days	Survived	Survived	Survived	Died, 1 h	Died, 36 h

*CVB* Coxsackievirus B, *NA* Not available, *DIC* Disseminated intravascular coagulation, *IVIG* Intravenous immunoglobulin

## Discussion

Manifestations of neonatal enterovirus infections range from asymptomatic, febrile illness to severe disease. A prospective cohort study in 2011–2012 in China reported 131 episodes of neonatal enterovirus infection (39.22%) among the 334 febrile neonates [72]. Xu et al. reported 16 cases of neonatal hand-foot-mouth disease with coxsackievirus A6 infection between 2016 and 2017 in Shanghai, China [73]. In this systematic review, a categorical summary of the most common complications of severe neonatal enterovirus infection was presented, including myocarditis, hepatitis, and encephalitis. In addition, the clinical features of other rare complications were reviewed. Lethality in each of the severe forms of neonatal enterovirus disease is high and varies between 11.5 and 38% depending on the type of disease. The most common severe complication was hepatitis, while myocarditis was the complication with the highest lethality rate. The age of onset in most neonates was less than 7 days and this might be related to the lack of specific transplacental neutralizing antibodies against the infecting serotype in newborns. The main sites of virus detection were the respiratory system, stools, cerebrospinal fluid, blood, and urine. Additionally, enteroviruses have also been detected in tissue samples from dead neonates, such as the heart, lung, spleen, and bone marrow. Isolation of the virus in the amniotic fluid or placenta as evidence of prenatal transmission has also been reported in severe cases. IVIG is frequently used for the treatment of enterovirus infections. Studies suggest that there may be more rapid clearance of viremia after IVIG therapy [74]. A retrospective study from Taiwan, China, evaluating the timing of IVIG administration revealed that early IVIG therapy (within 3 days of illness onset) may be beneficial for the survival of neonates with severe enteroviral infections [12]. The study analyzed 67 cases with culture-confirmed severe enteroviral infection. 41 infants (61%) received IVIG therapy and 29 cases were administered IVIG within 3 days of the illness onset with the mortality rate of 7% (2/29). While among the neonates with late IVIG therapy, the mortality rate was 50% (6/12). In our review, 40.9% of the neonates were treated with IVIG and the dosage of IVIG therapy varied from 0.5–2 g/kg/day. Therefore, more research is needed on the optimal dosage of IVIG treatment. Pleconaril, with activity against the picornaviruses, can bind to the viral capsid, thereby preventing viral uncoating within the cells [9]. A randomized, double-blind, placebo-controlled study of 43 enterovirus-infected neonates in America from 1999 to 2010 supported the potential efficacy of pleconaril [75]. The study enrolled 61 subjects (43 treatment, 18 placebo). Subjects in the treatment group received oral pleconaril of 5 mg/kg per dose 8 hourly for 7 days. Mortality rates in the treatment and placebo groups were 23 and 44%, respectively. They concluded that enterovirus-infected neonates in the treatment group had shorter times to culture, PCR negativity, and better survival.

Myocarditis is a common clinical manifestation of severe neonatal enterovirus infection, and is associated with a high lethality, reaching 38.6% in our review. The principal clinical manifestations of myocarditis include dyspnea, respiratory distress, apnea, arrhythmia, sudden cardiac arrest, and heart failure. Among them, there are a wide variety of arrhythmias, the most common being tachycardia, in addition to bradycardia, atrial flutter, ventricular fibrillation, and conduction block. CVB is the predominant enterovirus type causing myocarditis, especially CVB1 and 3. Weickmann et al. [25] reported a case of myocarditis caused by echovirus 6 in 2020, mainly manifesting as rare junctional ectopic tachycardia. Laboratory tests suggestive of myocarditis generally include elevation of troponin and BNP levels. Most electrocardiograms show myocardial ischemia, and a cardiac ultrasound often shows varying degrees of ventricular dilatation as well as decreased systolic function. We did not calculate the sensitivity and specificity of the diagnostic tests. Since most studies only report the positive result of the diagnostic test, the result of sensitivity and specificity might be overestimated. ECMO has been widely used in children with enterovirus myocarditis, and a total of 43 children who underwent ECMO were included in this study, with a survival rate of 39.5%. Furthermore, a study by Madden et al. [37] in 2011 that evaluated 24 neonates with enterovirus myocarditis receiving ECMO revealed that the survival rate was 33% with a high incidence of renal insufficiency in the children who died. In addition, heart transplantation and valve replacement have also been reported as successful treatments for enterovirus myocarditis.

Hepatitis or coagulopathy is the most common complication of severe neonatal enterovirus infection. It is also a serious complication of neonatal enterovirus infection with a high lethality rate (26.6%), and frequently co-occurs with myocarditis and encephalitis. The main body temperature abnormality in neonates with hepatitis is unstable body temperature, followed by fever. Other common clinical manifestations are rash, ascites, jaundice, hepatosplenomegaly, and thrombocytopenia. Hepatitis can further develop into acute liver failure, liver necrosis, fibrosis, and calcification. The vast majority of children develop the disease within 7 days. A high proportion of enteroviruses were found in the respiratory and stool samples. The proportion of CVB virus infections was high in neonates with hepatitis, with CVB1 and 3 being the most common, although there were also a few echovirus infections. The main supportive treatment for neonates with hepatitis is transfusion of blood components, such as platelets, plasma, and cryoprecipitate. Moreover, treatment with transfusion of maternal plasma has also been reported. Peritoneal dialysis, hemodialysis, blood replacement, and continuous venovenous hemofiltration have been applied in a small number of children; however, their therapeutic effect needs to be further studied.

There were some cases of neonatal enterovirus infection complicated by meningoencephalitis, and most cases had onset within 7 days. 15 cases of meningoencephalitis were accompanied with myocarditis and hepatitis. In these cases, meningoencephalitis was not the main complications or found at autopsy. The lethality rate of neonates with brain injury was low (11.5%), and the common sequelae among surviving neonates were hypotonia and cerebral palsy. Common clinical symptoms are fever and feeding difficulties. Neurological symptoms consist of epilepsy, irritability, hypotonia, and drowsiness. There was a report of a neonate who developed central diabetes insipidus. In most cases, the virus can be isolated from the cerebrospinal fluid and feces. In terms of virus typing, in addition to CVB and echovirus, a brain injury caused by EV71 was also reported. The most common abnormality in MRI is white matter injury. A case report by Hirata et al. [60] in 2011 concluded that diffusion-weighted imaging (DWI) was helpful for the early diagnosis of meningoencephalitis due to neonatal enterovirus infection.

Our article also reviewed some rare complications of neonatal enterovirus infection, such as HLH, pulmonary hemorrhage, pulmonary dysplasia, and bone marrow failure. All children developed the disease within 7 days, and there was a high proportion of mothers with antenatal fever and abdominal pain. Although these complications have only been reported in a few cases, the lethality rate is high and the disease progresses rapidly, often accompanied by damage to multiple organ systems.

However, there were several possible limitations to our study. The first limitation to this review was the small sample sizes within the included studies. Additionally, studies included were mainly case reports and case series, so we conducted a systematic review rather than a meta-analysis.

## Conclusion

Our study systematically reviewed the clinical characteristics of severe neonatal enteroviral infections. The results revealed that the lethality rate of severe neonatal enterovirus infections was high. Myocarditis was the complication with the highest lethality rate. The most common symptoms included temperature abnormalities, lethargy, rash, poor feeding, and respiratory symptoms. Additional clinical signs included arrhythmias, circulatory failure or shock, jaundice, and seizures. The main supportive treatment comprised transfusion of blood components, mechanical ventilation, and ECMO. Empirical antibiotics and IVIG were widely used. The antiviral medications used were pocapavir and pleconaril, although more clinical evidence regarding their efficacy is needed.

## Supplementary Information

**Additional file 1: Frame s1.** Searching strategy of Pubmed.

**Additional file 2: Table s1.** General characteristics of the included neonates.

**Additional file 3: Table s2.** Assessment of the risk of bias in included case reports.

**Additional file 4: Table s3.** Assessment of the risk of bias in included case series.

## Data Availability

All data generated or analysed during this study are included in this published article [and its supplementary information files].

## Abbreviations

CVB: Coxsackievirus B
EV71: Enterovirus 71
RT-PCR: Positive real-time polymerase chain reaction
JBI: Joanna Briggs Institute
DICD: Disseminated intravascular coagulation
AST: Aspartate aminotransferase
ALT: Alanine transaminase
IVIG: Intravenous immunoglobulin
BNP: Brain natriuretic peptide
ECMO: Extracorporeal membrane oxygenation
HLH: Hemophagocytic lymphohistiocytosis
DWI: Diffusion-weighted imaging
ECG: Electrocardiogram

## References

[1] CDC. Non-Polio Enterovirus. Available from: www.cdc.gov. Accessed 28 Nov 2020.

[2] Lugo D, Krogstad P (2016). Enteroviruses in the early 21st century: new manifestations and challenges. Curr Opin Pediatr.

[3] Noor A, Krilov LR (2016). Enterovirus infections. Pediatr Rev.

[4] Chuang YY, Huang YC (2019). Enteroviral infection in neonates. J Microbiol Immunol Infect.

[5] Marodi L (2006). Neonatal innate immunity to infectious agents. Infect Immun.

[6] JBI. Critical appraisal tools. Available from: https://jbi.global/critical-appraisal-tools. Accessed 28 Nov 2020.

[7] Le Van Quyen P, Desprez P, Livolsi A, Lindner V, Fafi-Kremer S, Helms P (2017). Peculiar clinical presentation of Coxsackievirus B4 infection: neonatal restrictive cardiomyopathy. Ajp Reports.

[8] Cantey JB, Hanners N, Mittal V (2012). A persistently fussy, febrile infant. Clin Pediatr (Phila).

[9] Bauer S, Gottesman G, Sirota L, Litmanovitz I, Ashkenazi S, Levi I (2002). Severe Coxsackie virus B infection in preterm newborns treated with pleconaril. Eur J Pediatr.

[10] Bersani I, Auriti C, Piersigilli F, Dotta A, Diomedi-Camassei F, Di Pede A (2020). Neonatal acute liver failure due to enteroviruses: a 14 years single NICU experience. J Matern Fetal Neonatal Med.

[11] Torres-Torres S, Myers AL, Klatte JM, Rhoden EE, Oberste MS, Collett MS, McCulloh RJ (2015). First use of investigational antiviral drug pocapavir (v-073) for treating neonatal enteroviral sepsis. Pediatr Infect Dis J.

[12] Yen MH, Huang YC, Chen MC, Liu CC, Chiu NC, Lien R, Chang LY, Chiu CH, Tsao KC, Lin TY (2015). Effect of intravenous immunoglobulin for neonates with severe enteroviral infections with emphasis on the timing of administration. J Clin Virol.

[13] Miyata I, Hanaoka N, Okabe N, Fujimoto T, Sakamoto S, Kasahara M, Saitoh A (2014). Echovirus 3 as another enterovirus causing life-threatening neonatal fulminant hepatitis. J Clin Virol.

[14] Pedrosa C, Lage MJ, Virella D (2013). Congenital echovirus 21 infection causing fulminant hepatitis in a neonate. BMJ Case Rep..

[15] Pino-Ramirez RM, Pertierra-Cortada A, Iriondo-Sanz M, Krauel-Vidal X, Munoz-Almagro C (2008). Neonatal echovirus 30 infection associated with severe hepatitis in twin neonates. Pediatr Infect Dis J.

[16] Ling LC, Pak CN, Chan PKS, Hiu LW, Cheng FWT, Tang JWT (2006). Probable intrafamilial transmission of coxsackievirus B3 with vertical transmission, severe early-onset neonatal hepatitis, and prolonged viral RNA shedding. Pediatrics..

[17] Tancabelic J, Haun SE (2004). Management of coagulopathy with recombinant factor VIIa in a neonate with echovirus type 7. Pediatric Blood Cancer.

[18] Wallot MA, Metzger-Boddien C, Auth M, Kehle J, Enders G, Dirsch O, Fiedler M, Voit T (2004). Acute liver failure associated with Coxsackie virus B2 infection in a neonate. Eur J Pediatr.

[19] Yen HR, Lien R, Fu RH, Chang LY (2003). Hepatic failure in a newborn with maternal peripartum exposure to echovirus 6 and enterovirus 71. Eur J Pediatr.

[20] Abzug MJ (2001). Prognosis for neonates with enterovirus hepatitis and coagulopathy. Pediatr Infect Dis J.

[21] Aradottir E, Alonso EM, Shulman ST (2001). Severe neonatal enteroviral hepatitis treated with pleconaril. Pediatr Infect Dis J.

[22] Ventura KC, Hawkins H, Smith MB, Walker DH (2001). Fatal neonatal echovirus 6 infection: autopsy case report and review of the literature. Modern Pathol.

[23] Wang J, Atchison RW, Walpusk J, Jaffe R (2001). Echovirus hepatic failure in infancy: report of four cases with speculation on the pathogenesis. Pediatr Dev Pathol.

[24] Konen O, Rathaus V, Bauer S, Dolfin T, Shapiro M (2000). Progressive liver calcifications in neonatal coxsackievirus infection. Pediatr Radiol.

[25] Weickmann J, Gebauer RA, Paech C (2020). Junctional ectopic tachycardia in neonatal enterovirus myocarditis. Clin Case Rep.

[26] Lee SR, Ko SY, Yoon SY, Lee YK, Shin SM (2019). Early detection and successful treatment of vertically transmitted fulminant enteroviral infection associated with various forms of arrhythmia and severe hepatitis with coagulopathy. Pediatric Infect Vaccine.

[27] Amdani SM, Kim HS, Orvedahl A, John AO, Said A, Simpson K. Successful treatment of fulminant neonatal enteroviral myocarditis in monochorionic diamniotic twins with cardiopulmonary support, intravenous immunoglobulin and pocapavir. BMJ Case Rep. 2018;2018:bcr2017224133. 10.1136/bcr-2017-224133. 10.1136/bcr-2017-224133 PMC5965763 29776940

[28] Cortina G, Best D, Deisenberg M, Chiletti R, Butt W (2018). Extracorporeal membrane oxygenation for neonatal collapse caused by enterovirus myocarditis. Arch Dis Child Fetal Neonatal Ed.

[29] McGovern E, Ryan E, McMahon CJ (2016). An infant with out-of-hospital cardiac arrest secondary to enteroviral myocarditis surviving up to cardiac transplantation. Cardiol Young.

[30] Morriss FH, Lindower JB, Bartlett HL, Atkins DL, Kim JO, Klein JM (2016). Neonatal Enterovirus infection: case series of clinical Sepsis and positive cerebrospinal fluid polymerase chain reaction test with myocarditis and cerebral white matter injury complications. AJP Rep..

[31] Bae EY, Lee EJ, Han SB, Lee JY, Jeong DC, Kang JH (2014). A case of myocarditis following neonatal meningitis caused by coxsackievirus B1 in spite of intravenous immunoglobulin treatment. J Trop Pediatr.

[32] Bissel SJ, Winkler CC, DelTondo J, Wang G, Williams K, Wiley CA (2014). Coxsackievirus B4 myocarditis and meningoencephalitis in newborn twins. Neuropathology.

[33] Bonnin A, Tassin M, Vauloup-Fellous C, Letamendia E, Stos B, Bonnet D, Gajdos V, Mabille M, Benachi A (2014). Case of a healthy infant born following antenatal enterovirus myocarditis and hydrops. J Clin Virol.

[34] Elisha N, Paret G, Rubinsthein M, Salem I (2013). Enteroviral myocarditis requiring extracorporeal membranous oxygenation in a 2 week old girl. Israel Med Assoc J.

[35] Schlapbach LJ, Ersch J, Balmer C, Pretre R, Tomaske M, Caduff R (2013). Enteroviral myocarditis in neonates. J Paediatr Child Health.

[36] Kobayashi D, Pettersen MD, Walters HL, Aggarwal S (2012). Mitral valve surgery for severe mitral regurgitation and dilated cardiomyopathy--a bridge to transplant: case report and a review of literature. Congenit Heart Dis.

[37] Madden K, Thiagarajan RR, Rycus PT, Rajagopal SK (2011). Survival of neonates with enteroviral myocarditis requiring extracorporeal membrane oxygenation. Pediatric Crit Care Med.

[38] Takahashi H, Tsukamoto K, Takahashi S, Nakamura T, Ito Y, Kaneko M, Sago H (2011). Reversible Atrioventricular block and Junctional ectopic tachycardia in Coxsackievirus B3-induced fetal-neonatal myocarditis without left ventricular dysfunction. AJP Rep.

[39] Freund MW, Kleinveld G, Krediet TG, van Loon AM, Verboon-Maciolek MA (2010). Prognosis for neonates with enterovirus myocarditis. Arch Dis Child Fetal Neonatal Ed.

[40] Al Senaidi K, Lacson A, Rebeyka IM, Mackie AS (2009). Echocardiographic detection of early myocardial calcification in acute neonatal myocarditis due to Coxsackie virus type B. Pediatr Cardiol.

[41] Meyer S, Gortner L, Gottschling S, Gartner B, Abdul-Khaliq H (2009). Cardiogenic shock in a neonate with enterovirus myocarditis. Klinische Padiatrie.

[42] Simpson KE, Hulse E, Carlson K (2009). Atrial tachyarrhythmias in neonatal enterovirus myocarditis. Pediatr Cardiol.

[43] Verma NA, Zheng XT, Harris MU, Cadichon SB, Melin-Aldana H, Khetsuriani N, Oberste MS, Shulman ST (2009). Outbreak of life-threatening coxsackievirus B1 myocarditis in neonates. Clin Infect Dis.

[44] Krogstad P, Hammon R, Halnon N, Whitton JL (2008). Fatal neonatal myocarditis caused by a recombinant human enterovirus-b variant. Pediatr Infect Dis J.

[45] Nathan M, Walsh R, Hardin JT, Einzig S, Connor BO, Balaguru D, Verma R, Starr JP Enteroviral sepsis and ischemic cardiomyopathy in a neonate: case report and review of literature. ASAIO J 2008;54(5):554–5, 555, DOI: 10.1097/MAT.0b013e3181877fc5. 10.1097/MAT.0b013e3181877fc5 18812754

[46] Simmonds J, Cubitt D, Ashworth M, Burch M (2008). Successful heart transplantation following neonatal necrotic enterovirus myocarditis. Pediatr Cardiol.

[47] Smets K, Keymeulen A, Wollants E, Lagrou K, Van Ranst M, Padalko E (2008). Detection of enteroviral RNA on Guthrie card dried blood of a neonate with fatal Coxsackie B3 myocarditis on day 17. J Clin Virol.

[48] Lu JC, Koay KW, Ramers CB, Milazzo AS (2005). Neonate with coxsackie B1 infection, cardiomyopathy and arrhythmias. J Natl Med Assoc.

[49] Inwald D, Franklin O, Cubitt D, Peters M, Goldman A, Burch M (2004). Enterovirus myocarditis as a cause of neonatal collapse. Arch Dis Child Fetal Neonatal Ed.

[50] Ouellet A, Sherlock R, Toye B, Fung KF (2004). Antenatal diagnosis of intrauterine infection with coxsackievirus B3 associated with live birth. Infect Dis Obstet Gynecol.

[51] Bendig JW, Franklin OM, Hebden AK, Backhouse PJ, Clewley JP, Goldman AP (2003). Coxsackievirus B3 sequences in the blood of a neonate with congenital myocarditis, plus serological evidence of maternal infection. J Med Virol.

[52] Murugan SJ, Gnanapragasam J, Vettukattil J (2002). Acute myocardial infarction in the neonatal period. Cardiol Young.

[53] Hoi-shan Chan S, Lun KS (2001). Ventricular aneurysm complicating neonatal coxsackie B4 myocarditis. Pediatr Cardiol.

[54] Oades PJ, Ladhani S (2015). Enteroviral meningoencephalitis in an infant: an increasingly recognised infection. Arch Dis Child.

[55] Guo SJ, Wang DX, Dai CL, Wu H. A neonate with hand, foot, and mouth disease complicated with brainstem encephalitis and pulmonary edema: a complete recovery. Pak J Med Sci. 2014;30(4):917–9. 10.12669/pjms.304.4528. 10.12669/pjms.304.4528 PMC4121726 25097545

[56] Ronellenfitsch S, Tabatabai J, Bottcher S, Diedrich S, Frommhold D, Schubert-Bast S (2014). First report of a Chinese strain of coxsackie B3 virus infection in a newborn in Germany in 2011: a case report. J Med Case Rep.

[57] Wu T, Fan XP, Wang WY, Yuan TM (2014). Enterovirus infections are associated with white matter damage in neonates. J Paediatr Child Health.

[58] Jones G, Muriello M, Patel A, Logan L (2015). Enteroviral Meningoencephalitis complicated by central diabetes Insipidus in a neonate: a case report and review of the literature. J Pediatric Infect Dis Soc..

[59] van den Berg-van de Glind GJD, de Vries JJC, Wolthers KC, Wiggers-de Bruine FT, Peeters-Scholte C, van den Hendef M (2012). A fatal course of neonatal meningo-encephalitis. J Clin Virol.

[60] Hirata O, Ishikawa N, Mizoguchi Y, Nakamura K, Kobayashi M (2011). A case of neonatal coxsackie B2 meningo-encephalitis in which serial magnetic resonance imaging findings reveal the development of lesions. Neuropediatrics..

[61] Brecht M, Jyoti R, McGuire W, Chauhan M (2010). A case of neonatal coxsackie B virus brainstem encephalitis. J Paediatr Child Health.

[62] Verboon-Maciolek MA, Groenendaal F, Cowan F, Govaert P, van Loon AM, de Vries LS (2006). White matter damage in neonatal enterovirus meningoencephalitis. Neurology..

[63] Miyoshi Y, Yoshioka S, Gosho H, Miyazoe S, Suenaga H, Aoki M, Hashimoto K (2020). A neonatal case of coxsackievirus B3 vertical infection with symptoms of hemophagocytic lymphohistiocytosis. Idcases..

[64] Watanabe Y, Sugiura T, Sugimoto M, Togawa Y, Kouwaki M, Koyama N, et al. Echovirus Type 7 Virus-Associated Hemophagocytic Syndrome in a Neonate Successfully Treated With Intravenous Immunoglobulin Therapy: A Case Report. Front Pediatrics. 2019;7 (no pagination)469. 10.3389/fped.2019.00469 PMC6867993 31799224

[65] Fukazawa M, Hoshina T, Nanishi E, Nishio H, Doi T, Ohga S, Hara T (2013). Neonatal hemophagocytic lymphohistiocytosis associated with a vertical transmission of coxsackievirus B1. J Infect Chemother.

[66] Lindamood KE, Fleck P, Narla A, Vergilio JA, Degar BA, Baldwin M, Wintermark P (2011). Neonatal enteroviral sepsis/meningoencephalitis and hemophagocytic lymphohistiocytosis: diagnostic challenges. Am J Perinatol.

[67] Orbach R, Mandel D, Lubetzky R, Ovental A, Haham A, Halutz O, Grisaru-Soen G (2016). Pulmonary hemorrhage due to Coxsackievirus B infection-a call to raise suspicion of this important complication as an end-stage of enterovirus sepsis in preterm twin neonates. J Clin Virol.

[68] Tassin M, Martinovic J, Mirand A, Peigue-Lafeuille H, Picone O, Benachi A, Vauloup-Fellous C (2014). A case of congenital echovirus 11 infection acquired early in pregnancy. J Clin Virol.

[69] Willems A, Benne CA, Timmer A, Bergman KA (2006). Fatal illness associated with pulmonary hypertension in a neonate caused by intrauterine echovirus 11 infection. Am J Perinatol.

[70] Tarcan A, Ozbek N, Gurakan B (2001). Bone marrow failure with concurrent enteroviral infection in a newborn. Pediatr Infect Dis J.

[71] Sauerbrei A, Gluck B, Jung K, Bittrich H, Wutzler P (2000). Congenital skin lesions caused by intrauterine infection with coxsackievirus B3. Infection..

[72] Lv XQ, Qian LH, Wu T, Yuan TM (2016). Enterovirus infection in febrile neonates: a hospital-based prospective cohort study. J Paediatr Child Health.

[73] Xu S, Li H, Qiao P, Xu G, Zhao D, Lin X, Qin Y, Yu H, Zhang X, Zhang W, Huang L (2020). Neonatal hand, foot, and mouth disease due to coxsackievirus A6 in Shanghai. BMC Pediatr.

[74] Yen MH, Tsao KC, Huang YC, Huang CG, Huang YL, Lin R, Chang ML, Huang CC, Yan DC, Lin TY (2007). Viral load in blood is correlated with disease severity of neonatal coxsackievirus B3 infection: early diagnosis and predicting disease severity is possible in severe neonatal enterovirus infection. Clin Infect Dis.

[75] Abzug MJ, Michaels MG, Wald E, Jacobs RF, Romero JR, Sanchez PJ (2016). A randomized, double-blind, placebo-controlled trial of Pleconaril for the treatment of neonates with Enterovirus Sepsis. J Pediatric Infect Dis Soc.

