# Supplementary material for: Clinical characteristics of severe neonatal enterovirus infection: a systematic review
Source: BMC Pediatr. 2021 Mar 15;21:127. doi: 10.1186/s12887-021-02599-y (PMC7958388; doi:10.1186/s12887-021-02599-y)
Supplement: Supplementary file 1 — Additional file 1: Frame s1. Searching strategy of Pubmed. [file 12887_2021_2599_MOESM1_ESM.docx]

Frame s1 searching strategy of Pubmed

#1 Enterovirus Infections [Mesh] OR Infections, Enterovirus OR Enterovirus Infection OR Infection, Enterovirus

#2 Coxsackievirus Infections [Mesh] OR Infections, Coxsackie Virus OR Coxsackievirus Infection OR Coxsackie Virus Infection OR Coxsackie Virus Infections OR Infections, Coxsackievirus

#3 Echovirus Infections [Mesh] OR Echo Virus Infections OR Infections, Echovirus OR Echo Virus Infection OR Echovirus Infection OR Infection, Echo Virus OR Infection, Echovirus OR Infections, Echo Virus

#4 #1 OR #2 OR #3

#5 Infant, Newborn [Mesh] OR Infants, Newborn OR Neonates OR Neonate OR Newborn OR Newborns OR Newborn Infants OR Newborn Infant

#6 #4 AND #5
