# Supplementary material for: Clinical characteristics of severe neonatal enterovirus infection: a systematic review
Source: BMC Pediatr. 2021 Mar 15;21:127. doi: 10.1186/s12887-021-02599-y (PMC7958388; doi:10.1186/s12887-021-02599-y)
Supplement: Supplementary file 2 — Additional file 2: Table s1. General characteristics of the included neonates. [file 12887_2021_2599_MOESM2_ESM.docx]

Table s1 General characteristics of the included neonates

| Reference | Country | Sample | Male/Female | GA (weeks) | BW (g) | Mode of delivery  Caesarean/ Vaginal | Maternal manifestation, days onset before delivery | Days at onset | Outcome, last follow-up |
| --- | --- | --- | --- | --- | --- | --- | --- | --- | --- |
| Miyata, 2014 | Japan | 1 | Female | 38 | 2372 | NA | Fever and diarrhea, 3 days | 3 | Survived, 68 days |
| Pedrosa, 2013 | Portugal | 1 | Male | 37 | NA | Caesarean | Fever, 0 | 5 | Died, 8 days |
| Torres-Torres, 2015 | America | 1 | Female | 33 | NA | Caesarean | Mild cough, 1 week; Febrile and tachycardic, 0 | 5 | Survived, 4 months |
| Yen, 2015 | Taiwan, China | 67 | Male/Female: 38/29 | Preterm/Full-term: 27/40 | 2500–3150 | Caesarean/Vaginal:34/33 | NA | <7: 58 | Survived/Died: 52/15 |
| Bersani, 2020 | Italy | 5 | NA | 36 | NA | NA | Fever and mild diarrhea, 2 months | 6 | Died, 27 days |
|  |  |  | Male | NA | NA | Caesarean | Fever and diarrhea, 6 weeks | 1 | Survived, 7 months |
|  |  |  | NA | 32 | NA | Caesarean | Febrile episode, 1 month | 1 | Died, 12 days |
|  |  |  | Male/Female: 1/1 | 35 | NA | NA | NA | 30 | Survived |
| Pino-Ramirez, 2008 | Spain | 1 | NA | 35 | NA | Caesarean | Flu-like syndrome, 1 week | 0 | Survived, 1 years |
|  |  | 1 | NA | 35 | NA | Caesarean | Flu-like syndrome, 1 week | 1 | Survived, 1 years |
| Rentz, 2006 | America | 1 | Female | 35 | NA | Caesarean | Severe abdominal pain, 0 | 6 | Died, 60 days |
| Ling, 2006 | Hong Kong, China | 1 | NA | Full-term | 3105 | NA | Fever with chills and rigors, 6 hours | 3 | Survived, 13 days |
| Wallot, 2004 | Germany | 1 | Male | 33 | 2110 | Caesarean | Gastrointestinal discomfort and diarrhea | 4 | Died |
| Tancabelic, 2004 | America | 1 | Male | 36 | 2300 | Vaginal | Febrile, 0 | 4 | Survived, 1 month |
| Yen, 2003 | Taiwan, China | 1 | Male | 35 | 2460 | Caesarean | Fever, 0 | 5 | Survived, 14 days |
| Bauer, 2002 | Israel | 2 | Female | 32 | 1735 | Caesarean | Flu-like illness, fever and myalgia, 1 week | 4 | Survived, 1 year |
|  |  |  | NA | 35 | 2215 | Caesarean | Fever, several hours | 5 | Survived |
| Ventura, 2001 | America | 1 | Male | Full-term | 3838 | Caesarean | Fever and an upper respiratory tract infection, 2 weeks | 4 | Died, 17 days |
| Konen, 2000 | Israel | 1 | Female | 32 | 1735 | Caesarean | Febrile disease with myalgia, a few days | 4 | Survived, 4 months |
| Wang, 2001 | America | 4 | Male | 37 | 2860 | NA | NA | 5 | Died |
|  |  |  | Male | 34 | NA | NA | NA | 4 | Died, 9 days |
|  |  |  | Male | 36 | 3600 | Vaginal | NA | 3 | Survived, 1 month |
|  |  |  | Male | 39 | 4200 | Vaginal | NA | 5 | Survived, 3 weeks |
| Aradottir, 2001 | America | 3 | Male | 37 | NA | Vaginal | Upper respiratory infection and myalgias, 1 week | 5 | Survived, 5 weeks |
|  |  |  | Female | Full-term | NA | NA | Slight congestion and fever, 1 week | 4 | Survived, 3 weeks |
|  |  |  | Male | 37 | NA | Vaginal | Lower abdominal pain and fever, several days | 4 | Died, 9 days |
| Abzug， 2001 | America | 16 | Male/Female: 6/10 | Preterm/Full-term: 8/8 | NA | Caesarean/Vaginal: 4/12 | Fever (n=8), abdominal pain (n=3), 2 weeks prepartum to 1 week postpartum (n=13) | 1-7 | Survived/Died: 11/5 |
| Weickmann, 2020 | Germany | 1 | Female | 37 | 2200 | Caesarean | NA | 11 | Survived, 3 months |
| Cortina, 2018 | Australia | 7 | Female | 38 | 3200 | Caesarean | NA | 5 | Survived, 21 months |
|  |  |  | Male | 36 | 2200 | Caesarean | NA | 7 | Died |
|  |  |  | Male | 38 | 2900 | Caesarean | NA | 6 | Died |
|  |  |  | Male | 36 | 3000 | Caesarean | NA | 4 | Survived, 54 months |
|  |  |  | Male | 37 | 2600 | Caesarean | NA | 5 | Died |
|  |  |  | Male | 40 | 3200 | Caesarean | NA | 8 | Survived, 34 months |
|  |  |  | Male | 39 | 3100 | Vaginal | NA | 8 | Died |
| Amdani, 2018 | America | 2 | Male | 37 | NA | Vaginal |  | 12 | Survived, 3 months (on 0.28mg/kg/day of enalapril) |
|  |  |  | Male | 37 | NA | Vaginal |  | 12 | Survived, 3 months (on 0.28mg/kg/day of enalapril) |
| McGovern, 2016 | Ireland | 1 | Female | NA | NA | NA | Sore throat and cough, 5 days | 13 | Survived, 3 months |
| Bonnin, 2014 | France | 1 | Male | 35 | 2380 | Caesarean | Fever and decreased fetal movements, 34 GA | 0 | Survived, 12 months |
| Bae, 2014 | Korea | 1 | Male | NA | NA | NA |  | 4 | Survived, 3 months (with left ventricular aneurism) |
| Schlapbach, 2013 | Australia | 2 | Female | 36 | NA | NA | NA | 8 | Died |
|  |  |  | Male | 38 | NA | NA | NA | 3 | Survived, 7 months |
| Elisha, 2013 | Israel | 1 | Female | Full-term | NA | NA | fever and nasal congestion, 0 day | 5 | Survived |
| Kobayashi, 2012 | America | 1 | Male | Full-term | 3600 | Vaginal | NA | 9 | Survived, 8 years (on medical therapy) |
| Takahashi, 2011 | Japan | 1 | Female | 38 | 3164 | Caesarean |  | 0 | Survived, 18 months |
| Madden, 2011 | America | 24 | Male/Female: 12/12 | NA | 2100-4500 | NA | NA | 7-15 | Survived/Died: 8/16 |
| Freund, 2010 | The Netherlands | 7 | Male | Full-term | NA | NA | Yes | 7 | Survived, 15.5 years (with severe DCM dilated cardiomyopathy, mild pulmonary hypertension) |
|  |  |  | Male | Full-term | NA | NA | Yes | 10 | Died, 3 weeks |
|  |  |  | Male | Full-term | NA | NA | None | 6 | Survived, 7.5 years (awaiting heart transplantation) |
|  |  |  | Female | Full-term | NA | NA | None | 7 | Survived, 3 years (with Moderate–severe DCM, aneurysm of LVPW) |
|  |  |  | Male | Full-term | NA | NA | None | 8 | Survived, 2 years (with mild DCM and MR mitral regurgitation) |
|  |  |  | Male | Full-term | NA | NA | Yes | 10 | Died, 7 weeks |
|  |  |  | Female | Full-term | NA | NA | Yes | 17 | Survived, 13 months (with moderate DCM) |
| Le Van Quyen, 2017 | France | 1 | Male | 37 | NA | NA | hyperthermia | 13 | Died, 17th day |
| Morriss, 2016 | America | 2 | NA | NA | NA | Vaginal |  | 7 | Survived, 9 months (with developmentally delayed) |
|  |  |  | NA | NA | NA | Caesarean | NA | 10 | Survived, 9 months |
| Bissel, 2014 | America | 2 | NA | 37 | NA | Caesarean | NA | 8 | Died, 8 days |
|  |  |  | NA | 37 | NA | Caesarean | NA | 10 | Died, 10 days |
| Lee, 2019 | Korea | 1 | Male | 37 | 3365 | Vaginal | mild cough and fever, 3 | 4 | Survived, 8 months |
| Meyer, 2009 | Germany | 1 | Female | NA | NA | NA | NA | 19 | Survived, 2 months |
| Al Senaidi, 2009 | Canada | 1 | NA | Full-term | NA | NA |  | 5 | Survived |
| Verma, 2009 | Amercia | 10 | NA | Preterm/Full-term: 5/5 | NA | Caesarean/Vaginal: 8/2 | Maternal illness (n=6) | 0-12 | Survived/Died: 9/1 |
| Simpson, 2009 | Amercia | 3 | Male | 36 | NA | NA |  | 0 | Survived, 24 days (with cardiac medication) |
|  |  |  | Female | 30 | NA | NA |  | 30 | Survived (with congestive heart failure with left ventricular dilation) |
|  |  |  | Female | 27 | NA | NA |  | 39 | Survived (with persistent moderate bi-atrial dilation) |
| Smets, 2008 | Belgium | 1 | Male | 39 | NA | NA |  | 17 | Died, 17 days |
| Simmonds, 2008 | UK | 1 | Female | 36 | 3530 | Caesarean |  | 5 | Survived, 1 year |
| Nathan, 2008 | Amercia | 1 | Male | NA | NA | NA |  | 8 | Survived (with cardiac medication and evaluation for cardiac transplantation) |
| Krogstad, 2008 | Amercia | 1 | Male | Full-term | NA | Vaginal |  | 3 | Died, 36 days |
| Lu, 2005 | Amercia | 1 | Male | 35 | 3035 | Caesarean | fever, 0 | 0 | Survived |
| Ouellet, 2004 | Canada | 1 | Male | 32 | 2380 | NA |  | 0 | Died, 24 hours |
| Inwald, 2004 | UK | 7 | Male/Female: 5/2 | NA | NA | Caesarean/Vaginal: 2/5 |  | 5-18 | Survived/Died: 4/3 |
| Bendig, 2003 | England | 1 | Female | Full-term | NA | Vaginal | fever, immediately postpartum | 0 | Died, 18 days |
| Murugan, 2002 | UK | 1 | Female | Full-term | NA | Vaginal |  | 9 | Survived, 9 months |
| Bauer, 2002 | Israel | 2 | Female | 32 | 1730 | Caesarean | influenza-like illness, fever and myalgia, 1 week | 4 | Survived, 1 year (with congestive heart failure) |
|  |  |  | NA | 35 | 2215 | Caesarean | fever, several hours | 5 | Survived |
| Chan, 2001 | Hong Kong, China | 1 | NA | 34 | 2380 | Vaginal | fever, headache, and myalgia, 1 week | 0 | Survived, 3 months (with ventricular aneurysm) |
| Guo, 2014 | China | 1 | Female | NA | NA | NA |  | 25 | Survived, 2 years |
| Wu, 2014 | China | 12 | Male/Female: 8/4 | Preterm/Full-term: 6/6 | 2450-4400 | Caesarean/Vaginal: 6/6 |  | <7: 11 | Survived: 12 |
| Ronellenfitsch, 2014 | Germany | 1 | Male | 38 | NA | Caesarean |  | 1 | Survived, 1 years (with muscle hypotonia) |
| Cantey, 2012 | America | 1 | Female | Full-term | 2852 | Caesarean | febrile, 48 hours | 4 | Survived |
| Brecht, 2010 | Australia | 1 | Female | Full-term | NA | Vaginal | upper respiratory tract infection, 1 week | 21 | Died, 43 days |
| Oades, 2015 | UK | 1 | Male | 37 | 3200 | NA | abdominal pain and an elevated CRP, 0 | 4 | Died, 7 days |
| Jones, 2013 | America | 1 | Male | 35 | 3200 | Caesarean | febrile illnesses, 1 week | 1 | Survived, 34 days |
| Hirata, 2011 | Japan | 1 | Female | 37 | NA | Caesarean | NA | 12 | Survived, 2 years (with severe neurological sequelae) |
| van den Berg-van de Glind, 2012 | The Netherlands | 1 | Female | 36 | NA | Caesarean | fever, 0 | 4 | Died, 15 days |
| Verboon-Maciolek, 2006 | The Netherlands | 6 | Female | 28 | 1100 | Vaginal |  | 55 | Survived, 6 years (with cerebral palsy, epilepsy, CVI cerebral visual impairment) |
|  |  |  |  | 36 | 2280 | Vaginal |  | 5 | Survived, 6 years (with cerebral palsy, epilepsy, BD behavioral disorder) |
|  |  |  |  | 40 | 3600 | Vaginal |  | 6 | Survived, 2 years |
|  |  |  |  | 33 | 1800 | Vaginal |  | 41 | Survived, 2 years |
|  |  |  |  | 40 | 3800 | Vaginal |  | 6 | Survived, 4 years |
|  |  |  |  | 33 | 2170 | Vaginal |  | 4 | Survived |
