# Supplementary material for: Clinical characteristics of severe neonatal enterovirus infection: a systematic review
Source: BMC Pediatr. 2021 Mar 15;21:127. doi: 10.1186/s12887-021-02599-y (PMC7958388; doi:10.1186/s12887-021-02599-y)
Supplement: Supplementary file 3 — Additional file 3: Table s2. Assessment of the risk of bias in included case reports. [file 12887_2021_2599_MOESM3_ESM.docx]

**Table s2 Assessment of the risk of bias in included case reports**

|  | Demographic characteristics | History | Current  clinical condition | Diagnostic tests | Intervention | Post-intervention clinical condition | Adverse events | Takeaway lessons | Overall appraisal |
| --- | --- | --- | --- | --- | --- | --- | --- | --- | --- |
| Bersani, 2020 | Yes | Yes | Yes | Yes | Yes | Yes | Unclear | Yes | Include |
| Torres-Torres, 2015 | Yes | Yes | Yes | Yes | Yes | Yes | Yes | Yes | Include |
| Miyata, 2014 | Yes | Yes | Yes | Yes | Yes | Yes | Unclear | Yes | Include |
| Pedrosa, 2013 | Yes | Yes | Yes | Yes | Yes | Yes | Unclear | Yes | Include |
| Pino-Ramirez, 2008 | Yes | Yes | Yes | Yes | Yes | Yes | Unclear | Yes | Include |
| Ling, 2006 | Yes | Yes | Yes | Yes | Yes | Yes | Unclear | Yes | Include |
| Rentz, 2006 | Yes | Yes | Yes | Yes | Yes | Yes | Unclear | Yes | Include |
| Tancabelic, 2004 | Yes | Yes | Yes | Yes | Yes | Yes | Unclear | Yes | Include |
| Wallot, 2004 | Yes | Yes | Yes | Yes | Yes | Yes | Unclear | Yes | Include |
| Yen, 2003 | Yes | Yes | Yes | Yes | Yes | Yes | Unclear | Yes | Include |
| Bauer, 2002 | Yes | Yes | Yes | Yes | Yes | Yes | Yes | Yes | Include |
| Aradottir, 2001 | Yes | Yes | Yes | Yes | Yes | Yes | Unclear | Yes | Include |
| Wang, 2001 | Yes | Yes | Yes | Yes | Yes | Yes | Unclear | Yes | Include |
| Ventura, 2001 | Yes | Yes | Yes | Yes | Yes | Yes | Unclear | Yes | Include |
| Konen, 2000 | Yes | Yes | Yes | Yes | Yes | Yes | Unclear | Yes | Include |
| Weickmann, 2020 | Yes | Yes | Yes | Yes | Yes | Yes | Unclear | Yes | Include |
| Lee, 2019 | Yes | Yes | Yes | Yes | Yes | Yes | Unclear | Yes | Include |
| Amdani, 2018 | Yes | Yes | Yes | Yes | Yes | Yes | Unclear | Yes | Include |
| Cortina, 2018 | Yes | Yes | Yes | Yes | Yes | Yes | Yes | Yes | Include |
| Le Van Quyen, 2017 | Yes | Yes | Yes | Yes | Yes | Yes | Unclear | Yes | Include |
| McGovern, 2016 | Yes | Yes | Yes | Yes | Yes | Yes | Yes | Yes | Include |
| Morriss, 2016 | Yes | Yes | Yes | Yes | Yes | Yes | Unclear | Yes | Include |
| Bae, 2014 | Yes | Yes | Yes | Yes | Yes | Yes | Unclear | Yes | Include |
| Bissel, 2014 | Yes | Yes | Yes | Yes | Yes | Yes | Unclear | Yes | Include |
| Bonnin, 2014 | Yes | Yes | Yes | Yes | Yes | Yes | Unclear | Yes | Include |
| Elisha, 2013 | Yes | Yes | Yes | Yes | Yes | Yes | Unclear | Yes | Include |
| Schlapbach, 2013 | Yes | Yes | Yes | Yes | Yes | Yes | Unclear | Yes | Include |
| Kobayashi, 2012 | Yes | Yes | Yes | Yes | Yes | Yes | Unclear | Yes | Include |
| Takahashi, 2011 | Yes | Yes | Yes | Yes | Yes | Yes | Unclear | Yes | Include |
| Freund, 2010 | Yes | Yes | Yes | Yes | Yes | Yes | Unclear | Yes | Include |
| Al Senaidi, 2009 | Yes | Yes | Yes | Yes | Yes | Yes | Unclear | Yes | Include |
| Meyer, 2009 | Yes | Yes | Yes | Yes | Yes | Yes | Unclear | Yes | Include |
| Simpson, 2009 | Yes | Yes | Yes | Yes | Yes | Yes | Unclear | Yes | Include |
| Krogstad, 2008 | Yes | Yes | Yes | Yes | Yes | Yes | Unclear | Yes | Include |
| Nathan, 2008 | Yes | Yes | Yes | Yes | Yes | Yes | Unclear | Yes | Include |
| Simmonds, 2008 | Yes | Yes | Yes | Yes | Yes | Yes | Yes | Yes | Include |
| Smets, 2008 | Yes | Yes | Yes | Yes | Yes | Yes | Unclear | Yes | Include |
| Lu, 2005 | Yes | Yes | Yes | Yes | Yes | Yes | Unclear | Yes | Include |
| Inwald, 2004 | Yes | Yes | Yes | Yes | Yes | Yes | Unclear | Yes | Include |
| Ouellet, 2004 | Yes | Yes | Yes | Yes | Yes | Yes | Unclear | Yes | Include |
| Bendig, 2003 | Yes | Yes | Yes | Yes | Yes | Yes | Unclear | Yes | Include |
| Bauer, 2002 | Yes | Yes | Yes | Yes | Yes | Yes | Yes | Yes | Include |
| Murugan, 2002 | Yes | Yes | Yes | Yes | Yes | Yes | Unclear | Yes | Include |
| Hoi-shan Chan, 2001 | Yes | Yes | Yes | Yes | Yes | Yes | Unclear | Yes | Include |
| Oades, 2015 | Yes | Yes | Yes | Yes | Yes | Yes | Unclear | Yes | Include |
| Guo, 2014 | Yes | Yes | Yes | Yes | Yes | Yes | Unclear | Yes | Include |
| Ronellenfitsch, 2014 | Yes | Yes | Yes | Yes | Yes | Yes | Unclear | Unclear | Include |
| Jones, 2013 | Yes | Yes | Yes | Yes | Yes | Yes | Unclear | Yes | Include |
| Cantey, 2012 | Yes | Yes | Yes | Yes | Yes | Yes | Yes | Yes | Include |
| van den Berg-van  de Glind, 2012 | Yes | Yes | Yes | Yes | Yes | Yes | Unclear | Yes | Include |
| Hirata, 2011 | Yes | Yes | Yes | Yes | Yes | Yes | Unclear | Yes | Include |
| Brecht, 2010 | Yes | Yes | Yes | Yes | Yes | Yes | Unclear | Yes | Include |
| Verboon-Maciolek, 2006 | Yes | Yes | Yes | Yes | Yes | Yes | Yes | Yes | Include |
