# Supplementary material for: Clinical characteristics of severe neonatal enterovirus infection: a systematic review
Source: BMC Pediatr. 2021 Mar 15;21:127. doi: 10.1186/s12887-021-02599-y (PMC7958388; doi:10.1186/s12887-021-02599-y)
Supplement: Supplementary file 4 — Additional file 4: Table s3. Assessment of the risk of bias in included case series. [file 12887_2021_2599_MOESM4_ESM.docx]

**Table s3 Assessment of the risk of bias in included case series**

|  | Criteria for inclusion | Condition measure | Condition identification | Consecutive inclusion | Complete inclusion | Demographic characteristics | Clinical information | Outcomes | Demographic information | Statistical analysis | Overall appraisal |
| --- | --- | --- | --- | --- | --- | --- | --- | --- | --- | --- | --- |
| Yen, 2015 | Yes | Yes | Yes | Yes | Yes | Yes | Yes | Yes | Yes | Yes | Include |
| Abzug, 2001 | Yes | Yes | Yes | Yes | Yes | Yes | Yes | Yes | Yes | Yes | Include |
| Madden 2011 | Yes | Yes | Yes | Yes | Yes | Yes | Yes | Yes | Yes | Yes | Include |
| Verma, 2009 | Yes | Yes | Yes | Yes | Yes | Yes | Yes | Yes | Yes | Yes | Include |
| Wu 2014 | Yes | Yes | Yes | Yes | Yes | Yes | Yes | Yes | Yes | Yes | Include |
